# Immune and Safety Analysis of *ultra*IPV^TM^, a Novel UVC-Inactivated Polio Vaccine

**DOI:** 10.3390/v17070915

**Published:** 2025-06-27

**Authors:** David A. MacLeod, John K. Tobin, Ruth V. Bushnell, Taralyn J. Wiggins, Shyamkumar TS, Ramchander Nadipelly, Steven Lawson, Viju V. Pillai, Gregory J. Tobin, Stephen J. Dollery

**Affiliations:** 1 Biological Mimetics, Inc., Frederick, MD 21702, USA; 2 Department of Radiation Oncology, University Hospitals, Case Western Reserve University, Cleveland, OH 44106, USA; 3 Department of Comparative Pathobiology, Purdue University College of Veterinary Medicine, West Lafayette, IN 47907, USA; 4 Department of Veterinary and Biomedical Sciences, South Dakota State University, Brookings, SD 57007, USA

**Keywords:** polio vaccine, UVC irradiation, *ultra*IPV, UV inactivation, IPV, UVC-inactivated polio vaccine, IPOL

## Abstract

The eradication of poliovirus remains a global health priority, with inactivated polio vaccines (IPVs) playing a pivotal role in immunization strategies. Over the past decades, advancements in IPV production have focused on optimizing safety, efficacy, and immunogenicity while addressing vaccine production and logistical challenges. This paper discusses a novel IPV candidate, *ultra*IPV^TM^, which departs from conventional formalin inactivation and uses a modern ultraviolet C (UVC) inactivation technology that includes a powerful antioxidant that protects virus epitopes from damage during and after irradiation. The potential of UVC inactivation to maintain structural integrity and immunogenicity of viral antigens, while circumventing safety issues with conventional vaccines, could bolster global polio eradication efforts and holds promise for applications to numerous other viral pathogens. Wistar rats were immunized with three dosages of *ultra*IPV^TM^, IPOL^R^, or vehicle alone. Immune responses were analyzed by whole-virus ELISA and antiviral neutralizing responses. Toxicity was analyzed primarily by increases in body weight and cytokine ELISA. Tolerability was analyzed by gross pathological and histological examinations. *ultra*IPV^TM^ was determined to be immunogenic and non-toxic. No pathological or histological abnormalities related to the vaccine were observed. The data suggest that *ultra*IPV^TM^ is immunogenic and well-tolerated in rats.

## 1. Introduction

Prior to the introduction of the inactivated (IPV) and oral (OPV) polio vaccines in the 1950s, poliomyelitis, also known as “infantile paralysis”, caused widespread flaccid paralysis and death, especially among immunologically naïve children [1]. The term poliomyelitis is derived from Greek and translates to gray (*polios*) marrow (*myelon*) which refers to the tissue in the center of the spinal cord, which when infected can cause severe and debilitating motor neuron dysfunction. Between 0.2 and 2% of unvaccinated children infected become paralyzed. In the US alone, annual polio cases numbered around 57,000 in 1952, with thousands left paralyzed. With the licensing of the first IPV by Dr. Jonas Salk in 1955 and OPV by Dr. Albert Sabin in 1961, and through aggressive vaccination campaigns, the number of polio cases plummeted. By the late 20th century, polio had been eliminated from most high-income countries, and vaccination efforts focused on low- and middle-income countries (LMIC). In 1988, there were approximately 350,000 cases worldwide when the Global Polio Eradication Initiative was launched [2]. Global eradication of wild-type poliovirus types 2 and 3 (WPV2 and WPV3) was certified by the World Health Organization (WHO) in 2015 and 2019, respectively [3]. WPV1 infections have been limited in both number and geographical region, with just a few remaining countries reporting infections since the start of 2017. OPV has been the workhorse of the eradication efforts and is both effective in stimulating robust immunity in most vaccinated people and inexpensive. Over the past 10 years, over 30 billion doses of OPV have been given to nearly three billion children worldwide [4].

Following oral administration, OPV primarily replicates in the gastrointestinal mucosa, where it stimulates both mucosal IgA and systemic IgG immune responses. Because of its attenuated phenotype, it cannot infect neural tissues to cause paralysis and is eventually eliminated by the body. However, in rare cases, the disease attenuation phenotype can be reversed during replication in the gut, and pathogenic virus can be shed. More rarely, individuals with primary immunodeficiencies such as severe combined immunodeficiency (SCID), common variable immunodeficiency (CVID), or agammaglobulinemia can develop immunodeficiency-associated vaccine-derived poliovirus (iVDPV), as their impaired immune systems fail to eliminate the attenuated virus, allowing prolonged viral replication and shedding. These people can excrete iVDPV for months or even years because they are unable to clear the viral infection. This prolonged replication period can contribute to the pool of neurotropic virus in the environment. Yet another risk presented by OPV is vaccine-associated paralytic poliomyelitis (VAPP) caused by mutation within the vaccine recipient or close contacts into a more neurovirulent form that mimics wild-type poliomyelitis in its clinical presentation [5]. Infection from all forms of vaccine-derived poliovirus (VDPV) has become an increasingly serious problem, and the majority of disease is now caused by VDPV [6]. Unlike OPV, IPV cannot cause VDPV.

Although the use of OPV supplanted its predecessor, IPV, as case numbers plummeted, the risks of VDPV eventually exceeded the risks of natural infection in many regions. Currently, a majority of countries have reinstated IPV and have far fewer VDPV cases, although virus surveillance is often lacking. Newer OPV derivatives are also being introduced that have been engineered to reduce the incidence of VDPV in regions with active infections. Novel OPVs (nOPV) composed of attenuated strains are engineered to be more resistant to reversion [7,8,9,10,11,12,13]. The nOPVs appear much safer but can still recombine with other circulating enteroviruses to become neuropathogenic in the field. There is ongoing debate about “gut” immunity and vaccination strategy, but most recognize that there will be a point after which a complete switch to IPV is an attractive option as eradication progresses and into the post-eradication era.

Conventional IPVs (cIPV) are formalin-fixed injectable products that stimulate robust systemic and some mucosal immunity in most vaccinated children [14,15,16,17]. The majority of IPV is produced by treating purified, wild-type, neuropathogenic virus for 2 to 4 weeks with formalin. IPV vaccines are more expensive than OPV and place significant burdens on poorly resourced nations and the agencies that help them. This has led to vaccination efforts with fractional doses of IPV as one approach [18,19]. The escape of polio virus from manufacturing plants into the environment has been documented and is an increasing concern as eradication proceeds [20,21,22]. Such a breach of biosecurity could undo eradication efforts and cost billions of dollars to recover. Because of the reservoir of virus in the environment and the rare chronic shedders, health authorities have planned to continue polio immunizations for at least two decades after WPV1 is certified to be eradicated [23,24,25,26]. Due to the continuing need for vaccines, the development of alternative, safer, and more affordable polio vaccines is ongoing.

Multiple strategies for producing safer polio vaccines have been underway for many years. Early efforts focused on substituting attenuated Sabin for neuropathogenic strains [27,28,29,30,31]. The Sabin-based IPVs (sIPV) reduce biosafety and biosecurity concerns during manufacturing. However, additional studies have shown that formalin inactivation damages the primary neutralization epitope located on VP1 of PV1 Sabin, which potentially reduces vaccine efficacy [32,33,34,35]. A more recent publication has demonstrated that sIPV stimulates statistically inferior neutralization responses to the PV1 component as compared to cIPV [36]. However, it is unknown whether this relatively small reduction in neutralizing antibodies would fail to protect in the real world. Based on these strategies, some countries, such as Japan and China, have approved formalin-inactivated Sabin vaccines, while others, such as the US, appear reluctant to do so [37,38,39,40,41,42]. Other strategies have explored the use of virus-like particles to make noninfectious immunogens [43,44,45,46].

To solve these problems, we have been developing an sIPV that is inactivated using a novel UVC-inactivation technology. Although irradiation inactivation of pathogens in the production of vaccines has been tested for decades, our novel vaccine candidate, *ultra*IPV^TM^, is produced using a powerful antioxidant complex that reduces destruction of epitopes during the inactivation process, as determined by mass spectroscopy. The complex was derived from *Deinococcus radiodurans*, an exceptionally radio-resistant bacterial species. *D. radiodurans* accumulates manganous peptide-phosphate complexes that protect internal enzymes, which can repair double-strand breaks and other damage caused by both radiation and desiccation. Our collaborator, Dr. Michel J. Daly (USUHS), and his colleagues discovered and developed the MDP (manganese-decapeptide-phosphate) complex, which protects the DNA repair machinery of *D. radiodurans*. Dr. Daly’s group also demonstrated that MDP protects the immunogenicity of gamma-irradiated viruses and bacteria [47,48,49,50,51]. Similar technologies are being developed for inactivated whole-cell vaccine candidates for Staphylococcus aureus (MRSA) and *Acinetobacter baumannii* [52,53,54]. Our initial IPV publication showed immunogenicity of gamma-irradiated PV1 and PV2 Sabin strains [55] Efforts to derive conditions to produce immunogenic PV3 Sabin using gamma irradiation were less successful, and we developed alternatives. The *ultra*IPV^TM^ candidate is produced from attenuated strains using MDP and UVC irradiation and stimulates similar levels of neutralizing antibodies in rats as IPOL^R^ and VeroPol^R^, two cIPV products [55,56]. The use of the MDP complex divorces undesirable oxidation of exterior proteins from damage to the viral RNA genome, which is desired for an inactivated whole-virus vaccine. The candidate has several advantages when compared to cIPV. In addition to reduced biosafety and biosecurity concerns during manufacturing, the inactivation process takes seconds instead of weeks. More importantly, perhaps, the UVC-MDP method causes less damage to immunogenic epitopes and, therefore, results in far more doses per milligram of stock virus, as shown in Tobin et al., 2024. The improved yield can alleviate global shortages of IPV while reducing production costs [56]. Previous analyses of freeze-thaw stability suggest that *ultra*IPV^TM^ could also be used to supply long-term strategic reserves in case of the re-emergence of polioviruses long after eradication.

As part of the *ultra*IPV^TM^ development process, we have performed a pre-GLP analysis of immunogenicity, toxicity, and tolerability. Wistar rats, a well-accepted model for polio vaccines, were immunized intramuscularly every 14 days. The rat groups were immunized with (1) vehicle only, (2) low-dose *ultra*IPV^TM^, (3) medium-dose *ultra*IPV^TM^, (4) high-dose *ultra*IPV^TM^, and (5) IPOL^R^ (Sanofi Winthrop, Bridgewater, NJ, USA) as a comparator. The three doses were used to determine a concentration effect, if toxicity or adverse reactions were observed. Both 3 and 21 days after the fourth immunization, 50% of the rats from each group were humanely euthanized and tissues collected for analysis, which included analysis of overall immune responses and neutralizing antibodies, selected cytokines, body weights, gross pathology, and inflammatory responses in tissues detected by histopathology. No significant differences were detected between the high-dose *ultra*IPV^TM^ group and IPOL^R^, which both included dose-matched components of PV1, PV2, and PV3 viruses.

## 2. Materials and Methods

### 2.1. Virus Production and Purification

PV1-Sabin, nOPV2, and PV3-Sabin viruses were propagated on H1 HeLa suspension cells in SMEM supplemented with 10% fetal bovine serum, a solution of penicillin and streptomycin (100 U/mL final concentration), nonessential amino acids (1×) (GIBCO 11140-050, Carlsbad, CA, USA), D-Glucose (4 g/L) (Gibco A2494001, Carlsbad, CA, USA), L-Glutamine (4 mM) (Gibco 25030081, Carlsbad, CA, USA), and Poloxamer 188 (0.1%) (Gibco 24040032, Carlsbad, CA, USA) using standard procedures [55,56]. Briefly, cells were pelleted at 2000× *g* when the growth density achieved 1.2–1.5 × 10^6^ cells per mL. The cells were resuspended in VaccineXpress cell (Cytiva HyClone SH31126.01, Logan, UT, USA) without serum (but still all the additional supplements at previously stated concentrations) at a density of 1 × 10^8^ c/mL and virus stock added at a multiplicity of infection of 5–10 TCID_50_ per cell. After a 1-h virus attachment period, the sample was diluted to a density of 1 × 10^7^ c/mL using the same media as previously mentioned and shaken at 200 rpm at 37 °C for 10 h for PV1, 8 h for PV2, and 12 h for PV3. After the infection process, the cells were pelleted, freeze-thawed three times, and clarified of cell debris by centrifugation at 5000× *g* for 30 min. Cellular macromolecules were precipitated using domiphen-bromide and removed by centrifugation at 20,000× *g* for 20 min. The viruses were pelleted through a pad of 30% sucrose (*w*/*w*) by ultracentrifugation at 100,000× *g* for 6 h and resuspended in a small volume of PBS. 1 M MES pH 6.0 buffer was added to a final concentration of 100 mM and the virus pumped through a small column of Toyo Pearl Sulfo-650F cation exchange resin (Tosoh Biosciences, San Francisco, CA, USA). After washing with 100 mM MES pH 6.0, the virus was eluted using a step gradient of increasing NaCl concentrations. Western blots were used to identify peak fractions, which were pooled, washed, and concentrated in 100 kDa molecular weight cut-off spin cartridges (Millipore, Saint Louis, MO USA), and quantified for total protein using a Coomassie-based assay and for virus concentration using western blots with purified virus standards that had been previously characterized by mass spectroscopy.

### 2.2. UVC Inactivation of Polio Viruses

Then, 0.1–0.3 mg/mL of PV1-S, nOPV2, and PV3-S were formulated with the MDP complex consisting of 25 mM potassium phosphate buffer (pH 7.4), 2.5 mM MnCl_2_, and 3 mM decapeptide (DP1: DEHGTAVMLK) as previously described [56]. The MnCl_2_ was added last using a 30 mM stock to reduce the chances of precipitation with phosphate. MDP-virus samples were placed into thin-wall 0.2 mL PCR tubes, ambient air was purged with argon, and the tubes were placed onto a Model UVP UVG-54 UVC wand (Analytik Jenna US, Upland, CA, USA) outputting 4.5 mWatts/cm^2^. UVC output was measured using a UV512C digital light meter (General Tools and Instruments, LLC, Secaucus, NJ, USA). Samples were exposed for 30 s to receive approximately 135 mJoules of light energy. The D antigen concentration of the three viruses was determined by a WHO-standardized ELISA [56].

### 2.3. Rat Immunization 

Animal studies were performed under humane conditions using protocols approved by the Institutional Animal Care and Use Committee at South Dakota State University (IACUC protocol number 2302-023A, approved 22 February 2023). Wistar rats, a widely accepted animal model for polio vaccine analysis, between 6 and 8 weeks of age and of equal numbers of males and females were used for immunization studies. Animals were housed in a pathogen-free facility and observed twice daily to assess potential health problems. Rats were immunized by intramuscular injection into the quadriceps without adjuvant on Days 0, 13, 26, and 39. The approved dose strength for conventional IPVs is 40:8:32 D antigen units of the PV1, PV2, and PV3 components, respectively. The *ultra*IPV^TM^ candidate antigen was purposely formulated in M-199 buffer without adjuvant, as is done with IPOL, to facilitate comparison. Group 1 rats received vehicle only (M-199 buffer). Groups 2, 3, and 4 were immunized with low, medium, and high doses of inactivated viruses consisting of 10:5:10, 20:10:20, 40:20:40 and D antigen units, respectively. D antigen concentrations were determined using a standardized D ELISA with Sabin-specific antibodies [57]. The dose compositions were selected, in part, based on neutralization titers of young children immunized with Sabin-based IPV [36]. Group 5 rats were immunized with a standard human dose of IPOL^R^ (Sanofi) as a comparator, which consists of 40:8:32 DU of the three viruses. The standard human dose is formulated in a volume of 0.5 cc, which was split into two equal injections in the rats. The rats were weighed weekly. Four days and twenty days after the final immunization, 50% of the rats from each group were anesthetized and humanely euthanized for analysis of acute and chronic toxicity, respectively. Analyses included serology, gross pathology, and histopathology. A pre-immunization blood sample was collected on Day 1, an interim sample on Day 38, and final bleeds during euthanasia.

### 2.4. Analysis of Immune Responses: Total Binding Antibodies to Polioviruses, Neutralization Titers, and Responses to the Decapeptide

Serum samples were assessed for total binding antibodies, antiviral neutralizing antibodies, and antibodies to the decapeptide, which was not removed prior to immunization. Total binding antibodies to the three virus components were assessed separately by ELISA using 10 ng of purified virus bound overnight to flat-bottom high-binding ELISA plates in water. 0.1 mL serum samples were diluted 1:100 in PBS and incubated at room temperature for 1 h using quadruplicate wells. The plates were washed four times with PBS containing 0.1% Tween-20 detergent and then probed with goat anti-rat secondary antibody conjugated to horseradish peroxidase. After a 1 h incubation, the plates were washed and developed using TMB reagent. The colorimetric reactions were stopped with 1.0 N H_2_SO_4_, and the plates were read at A450 nm.

Serum samples were tested for antiviral neutralizing antibodies using standard methods established by the WHO Expert Committee on Biological Standardization [58]. Briefly, 350 µL volumes of 2-fold virus dilutions were incubated separately with 700 TCID_50_ of each titered virus for 1 h at room temperature. 25 µL of virus-serum mixtures were applied to 96-well plates of MRC-5 indicator cells for 1 h at room temperature using six replicate wells. The plates were washed with PBS, media replaced, and then incubated for 5 days at 37 °C in a humidified incubator with 5% CO_2_. Each well was scored as either infected or uninfected using a phase-contrast microscope at 100× and 300× magnification. The neutralization titers were calculated using a standard Spearman–Kärber method.

To determine whether the animals raised antibodies to the DP1 peptide that was included in the inactivated vaccine candidate, a peptide ELISA was performed. Briefly, 96-well plates were coated with 10 ng of DP1 peptide per well overnight at room temperature. Sera was analyzed in pools by group and split up based on euthanization date (five animal groups, ten pools). Then, 100 µL volumes of 1:300 and 1:1000 dilutions of sera were applied to the wells using six replicates. After 2 h incubation at room temperature, the wells were washed four times with PBS containing 0.1% Tween-20 and probed with goat anti-rat secondary antibody conjugated to horseradish peroxidase. After a one-hour incubation, the plates were washed and developed using TMB reagent. The colorimetric reactions were stopped with 1.0 N H_2_SO_4_, and the plates were read at A450 nm. Results are shown in Supplementary Figure S1.

### 2.5. Histology

Following euthanasia, tissues were examined for signs of gross pathology abnormalities by a board-certified veterinary pathologist (Co-author Viju Pillai, PhD, DVM). Tissue samples were then removed, fixed in 4% formaldehyde, embedded in paraffin blocks, and thin sectioned (4 microns). Sections were stained with hematoxylin and eosin using standard techniques. Briefly, sections on glass slides were deparaffinized and rehydrated using xylene and graded ethanol (100%, 95%, 80%) and hydrated in water. The slides were immersed in hematoxylin for 3 min, followed by several water rinses. The slides were counterstained with 1% eosin in water for 3 min and then dehydrated with three washes of five minutes each in 95% ethanol, 100% ethanol, and Xylene. After covering the slides with slide covers, the sections were examined under phase-contrast light microscopy. Digital images were collected, and tissue abnormalities were carefully recorded.

### 2.6. Statistical Analyses

ELISA absorbances were compared using Kruskal–Wallis and Dunn’s test with Benjamini–Hochberg correction for multiple comparisons. Analyses were performed using Python v3.11.5 with Pandas v2.0.3, statsmodels v0.14.0, and SciPy v1.11.1 libraries. Viral neutralization titers were plotted on log-2 scales and analyzed using Prism Version 8.4.2 (679). Histology analysis and heatmap were generated with Python v3.11.5, Matplotlib v3.7.2, and Seaborn v0.12.2. Cytokine serum ELISA data were plotted and analyzed with Python v3.11.5 using NumPy v1.24.3, Pandas v2.0.3, and Matplotlib v3.7.2.

## 3. Results

**Figure 1:**
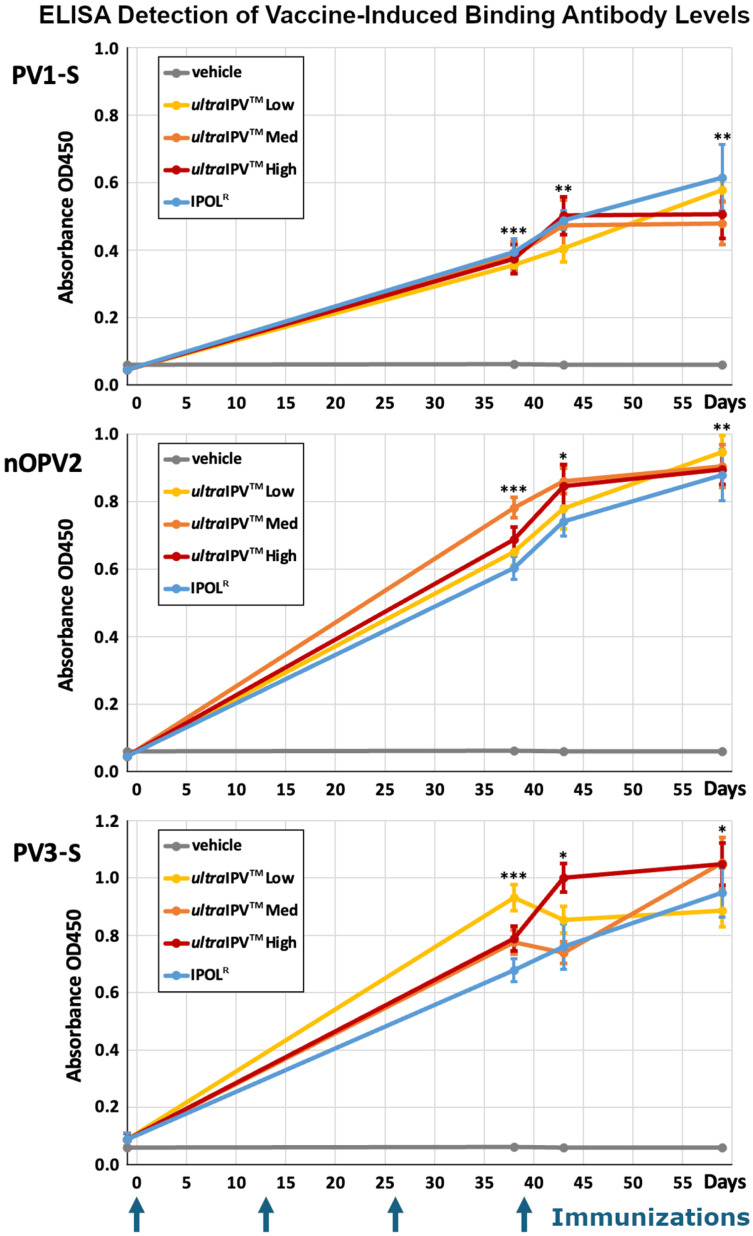
Virus-Binding Antibody Levels Induced by Vaccine. Arrows indicate vaccine immunization timepoints. Mean absorbances shown; error bars represent SEM. * *p* < 0.05; ** *p* < 0.01; *** *p* < 0.001 for all vaccine groups compared to vehicle control.

As a first step toward examining the immune responses to vaccination, we analyzed virus-binding antibodies using an ELISA approach in which the three virus components were bound to separate plates. Serum samples from test bleeds taken prior to the first (Day 1) and third (Day 38) immunization and upon euthanasia (Days 43 and 59) were analyzed. The sera from individual rats were tested for antibodies against the three serotypes of polio using a 1:100 dilution. These were compared to a group immunized with the commercial IPOL^R^ vaccine and a vehicle-only control. The graphs in Figure 1 plot the mean OD450 values for each group.

The pre-immune serum exhibited negligible antigen binding, as would be expected. Then, at all post-immunization timepoints, all serum from animals vaccinated with either IPOL^R^ or any of the three dose levels of *ultra*IPV^TM^ showed antibody levels significantly greater than in serum from vehicle controls. The established IPOL^R^ vaccine and all three doses of the novel *ultra*IPV^TM^ vaccine candidate were seen to perform similarly in inducing roughly equivalent levels of anti-polio antibodies. Notably, at each timepoint, no vaccine preparation exhibited any significant difference from any other in the levels of anti-polio antibodies elicited (see Supplementary Figure S2).

**Figure 2:**
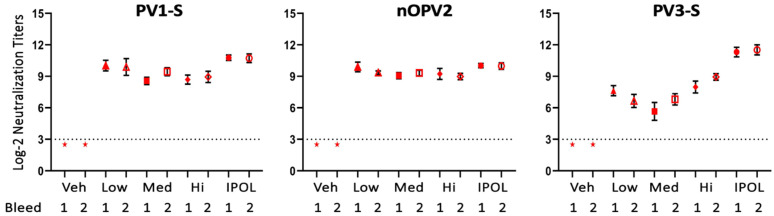
Virus Neutralization Titers. Veh = vehicle, Low = *ultra*IPV^TM^ Low, Med = *ultra*IPV^TM^ Med Hi = *ultra*IPV^TM^ High, IPOL = IPOL^R^ Bleed #1 was performed 4 days after 4th immunization, and bleed #2 was at 20 days after 4th immunization. ★ = below the threshold of detection. Error bars show standard deviation.

Given that *ultra*IPV^TM^ at even the lowest dose induced comparable levels of anti-polio antibodies to the other groups, we then sought to assay the functional effectiveness of these antibodies at neutralizing viral strains. Virus-neutralizing titers of 1:8 (indicated by the dotted lines) are the accepted correlates of immune protection from infection in humans. Neutralization assays were performed with serum collected from two timepoints post-vaccination. Similar neutralization titers were seen for PV1-S and nOPV2, between all *ultra*IPV^TM^ doses and IPOL^R^, whereas for PV3-S, the IPOL^R^ preparation showed somewhat higher titers (Figure 2). The data show that all rats developed antiviral titers that correlate with protection (1:8 or 2^3^).

**Figure 3:**
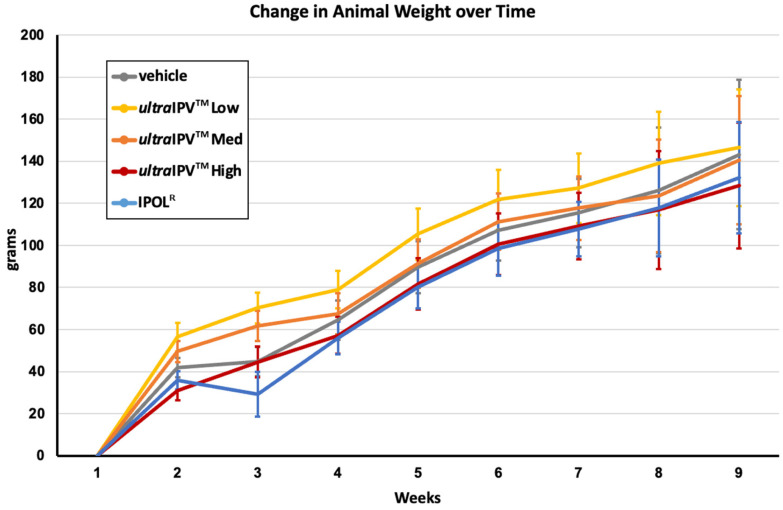
Change in Animal Weight over Time. Mean change in animal weights from baseline (Week 0) is shown; error bars represent SEM.

We then proceeded to test whether rats immunized with *ultra*IPV^TM^ exhibited overt signs of adverse reactions to vaccination. As a general proxy for animal health, the weight of the animals was recorded for each group over the course of the experimental protocol. As seen in Figure 3, animals in all vaccination groups as well as the vehicle controls showed weight gain in a similar range over the 8-week study. There was a slight decrease in weight in the IPOL^R^ group at week 2, but the animals recovered swiftly. No *ultra*IPV^TM^ vaccinated group showed any signs of retardation of the expected growth, and there was no indication of any safety concern from the novel vaccine preparations by this metric.

**Figure 4:**
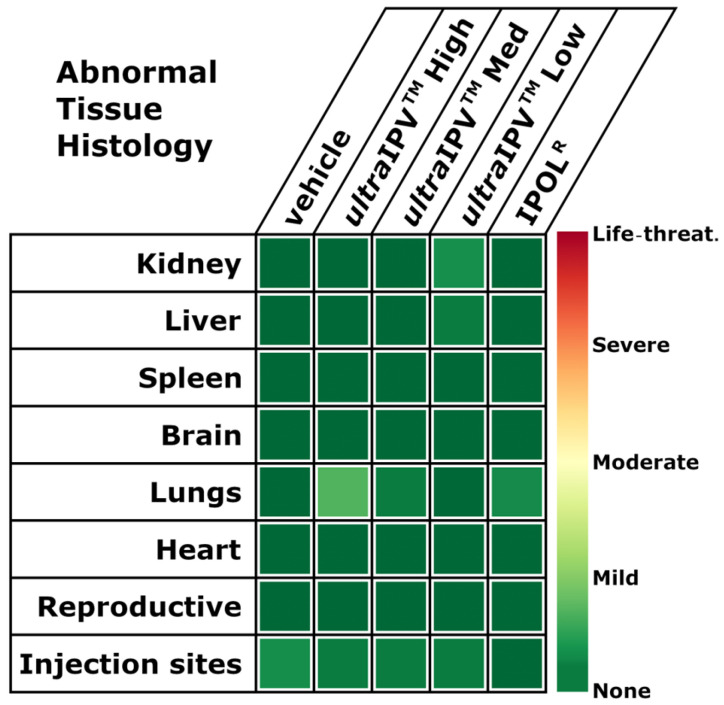
Tissue Histology Analysis. Mean tissue abnormality scoring by severity from none to life-threatening.

For a more comprehensive view of animal health in response to vaccination, histological analysis was performed across multiple tissue types in different organ systems. Tissues were scored for abnormalities, in particular for signs of inflammation and/or immune cell infiltration. Figure 4 shows a summary heatmap of the fraction of animal samples in each group of 12 rats. Most tissue samples were normal for every group of animals. The most common non-normal observation was rare or mild neutrophile infiltration in lungs (high-dose *ultra*IPV^TM^ group) and mild mononuclear cell infiltrates at the injection sites (Vehicle alone group). One rat from the IPOL^R^ immunization group had a focus of moderate numbers of mature neutrophiles surrounding a larger caliber vessel in the brain. For the full tissue scoring table with specific annotations, see Supplementary Figure S3.

**Figure 5:**
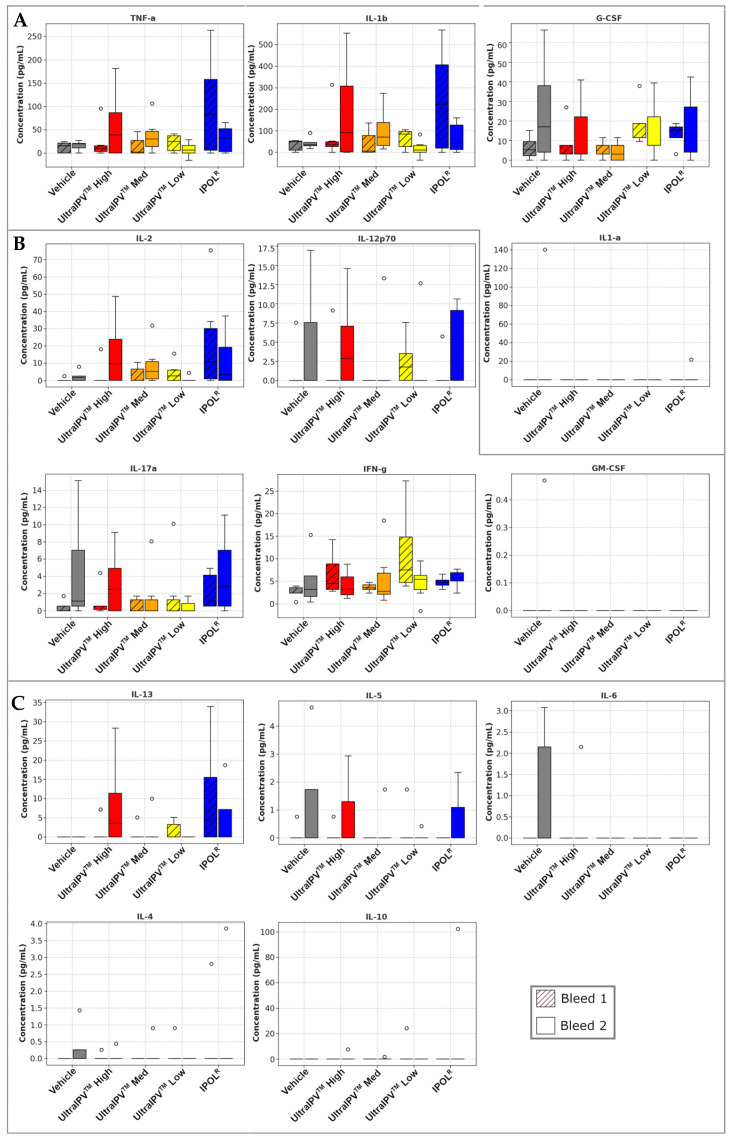
Serum Cytokine Panel. Median serum concentration of cytokines shown, grouped loosely into (**A**) proinflammatory cytokines of primary concern for safety analysis, and those mainly involved in mediating (**B**) T cell response, and (**C**) B cell response. Box height represents interquartile range (IQR); whisker bars show minimum and maximum data points within 1.5 × IQR from 1st and 3rd quartiles, respectively; circles show outlier data points defined as being >1.5 × IQR from middle 50% of values.

As an additional assessment of vaccine safety and tolerability, serum samples from immunized animals were assayed for cytokine concentrations. These measurements would reveal any systemic inflammation or adverse reactions to immunization, such as cytokine storm. Results are shown in Figure 5. No cytokine assayed was above reference ranges for healthy animals. In some cases, the levels of cytokines were below the threshold of detection, which can be expected in a broad panel. Although many of these cytokines can contribute directly or indirectly to diverse immune responses, we grouped them loosely by primary function. In Figure 5A, levels of proinflammatory cytokines of main concern for safety, and which could indicate systemic inflammation, were not elevated with *ultra*IPV^TM^ immunization. Indeed, for TNF-α and IL-1β, levels trended higher in the IPOL group. TNF-α is produced primarily by activated macrophages and is a well-known mediator of local and systemic inflammatory responses [59]; its serum levels are often reported to increase post-vaccination [60]. IL-1β promotes the production of other proinflammatory cytokines, causes proliferation of T and B cells, and can stimulate the production of antibodies [61,62]. G-CSF is an important regulator of innate immunity by stimulating the production and mobilization of neutrophils [63]. Elevated levels of G-CSF have been reported as associated with adverse local reactions in testing of a recombinant varicella-zoster (shingles) vaccine [64]. Figure 5B shows cytokines that are primarily involved in the T cell response to vaccination. None of these were elevated to a degree that would be at all alarming. Il-2 and Il-12p70 stimulate the proliferation and differentiation of T cells, and they enhance the cytotoxicity of CD8* T cells and NK cells [65,66]. IFN-γ performs diverse functions pivotal to immunity, among them enhancing antigen presentation by upregulating MHC class I and II expression, activating macrophages, promoting Th1 differentiation, and exerting a concerted battery of antiviral and antitumor responses [67]. Figure 5C shows cytokines important for B cell maturation and antibody production. Of these, IL-5 and especially IL-13b can potentially contribute to adverse reactions to vaccines when levels are elevated far above reference ranges [68], but that was not observed here. Indeed, the IPOL group showed a trend toward higher levels than the *ultra*IPV^TM^ groups. Il-13 is a key mediator of allergic reactions. It promotes B cell maturation and IgE class switching, which can lead to allergic inflammation. It also stimulates fibroblast proliferation, which can cause fibrosis seen in chronic inflammation [69].

## 4. Discussion

The development of novel vaccines as alternatives for conventional IPV and OPV vaccines is essential for maintaining continued immune pressure to prevent the re-emergence of polio viruses. The use of Sabin-based or other attenuated strains in inactivated polio vaccines greatly enhances biosafety and biosecurity during manufacturing. These advantages may allow additional companies or countries to enter the manufacturing market and decentralize vaccine production. The use of UVC to inactivate the viruses in the presence of the MDP antioxidant complex resulted in an unanticipated level of epitope protection, which could enable more doses to be manufactured per milligram of input virus [56]. This is a second feature that may further decentralize vaccine production, because culture facilities sized for producing 1 million doses may now be able to produce 10 million or more doses.

The studies presented in this report demonstrate that the *ultra*IPV^TM^ candidate is relatively non-toxic and compares favorably to the current vaccine IPOL in terms of risk proxies and immunogenicity. Total binding antibodies induced by the three *ultra*IPV^TM^ groups closely tracked IPOL^R^ as shown in Figure 1. Antiviral neutralization titers were similar for the PV1 and PV2 components. Unexpectedly, neutralization titers for the PV3 component were lower than as seen with IPOL^R^. This has not been the case in several prior immunization studies [56] and will require additional follow-up. Upon euthanasia, no gross pathological abnormalities were found in any groups, and histological analysis showed no signs of adverse reactions to the vaccine candidate. Interestingly, the histology slides showed some signs of mild inflammation in the Group 1 (Vehicle only) rats at the site of injection. In addition, five of the twelve rats from the high-dose of *ultra*IPV^TM^ showed rare (two rats), mild (two rats), or moderate (one rat) infiltrates of neutrophils surrounding vessels in lung sections (Figure 4). Weight gains were similar across the groups, with a slight lag for the IPOL^R^ comparison group, which was generally not statistically lower than the low-dose *ultra*IPV^TM^ group. Each rat serum sample was analyzed for 14 cytokines. Figure 5 shows the statistical analysis of the five groups analyzed for acute and chronic responses. Although it is not thoroughly clear whether select cytokine responses are desired or undesired, the two IPOL^R^ sub-groups generally presented the highest concentrations of proinflammatory cytokines, suggesting that this well-established vaccine stimulates inflammatory responses that are well-tolerated.

Taken together, the data show that the *ultra*IPV^TM^ candidate was similarly immunogenic as the IPOL^R^ comparator with the exception of diminished anti-PV3 neutralization titers, which were not observed in previous rat immunization studies [56]. The similarities in anti-PV1 and anti-PV2 neutralization titers between the low, medium, and high dose groups, which closely follow previously reported results [56], suggests that a lower dose may be recommended in future studies. The reduced anti-PV3 neutralization titer suggests that a higher dose may be used in the future. One rat had a focus of moderate numbers of mature neutrophils surrounding a larger caliber vessel in the brain, and it is unclear whether this is related to the vaccine or a rare development in a random animal. A minority of the rats in the high-dose *ultra*IPV^TM^ group demonstrated rare to moderate infiltrates of mature neutrophils surrounding vessels in the lung. We have not previously seen visible health changes in the more than 100 rats immunized with *ultra*IPV^TM^ throughout our studies, and we will examine this more closely in a follow-up study.

### Future Implications

We conclude that the *ultra*IPV^TM^ candidate is immunogenic and largely well-tolerated in Wistar rats. The MDP-UVC inactivation method is less damaging to immunogenic epitopes than formalin inactivation, and this platform could be used to generate vaccine candidates for a wide variety of pathogens.

## Data Availability

All data from this study are contained within this publication.

## Supplementary Materials

The following supporting information can be downloaded at: https://www.mdpi.com/article/10.3390/v17070915/s1. Figure S1. DP1 ELISA. A450nm absorbances shown for ELISA assay that was performed to determine whether sera from immunized rats reacted to the DPI peptide required in the inactivation step of *ultra*IPV^TM^ preparation. Figure S2. Binding Antibody ELISA Statistical Analysis. Differences in ELISA absorbances were tested for significance using Kruskal-Wallis and Dunn’s test with Benjamini-Hochberg correction for multiple comparison; corrected p values are shown. Figure S3. Tissue Histology Scoring. Histological analysis scores represent the severity of tissue sample abnormalities for individual animals in each vaccination group.

## Abbreviations

The following abbreviations are used in this manuscript:

°C	Degrees in Celsius
*g*	gravitational force used in centrifugation
IPV	Inactivated polio vaccine
MDP	Manganese-decapeptide-phosphate antioxidant complex
MES	2-(N-morpholino)ethanesulfonic acid buffer
NaCl	Sodium chloride
OPV	Oral polio vaccine
PBS	Phosphate-buffered saline
PV1	Poliovirus serotype 1
RNA	Ribonucleic acid
sIPV	Sabin-based IPV
SMEM	Spinner modification of minimum essential Eagle’s medium
UVC	Ultraviolet light in the C band (254 nm)
VAPP	Vaccine-associated paralytic poliomyelitis
VDPV	Vaccine-derived poliovirus
WHO	World Health Organization
WPV1	Wild-type poliovirus serotype 1
