# Supplementary material for: Immune and Safety Analysis of ultraIPVTM, a Novel UVC-Inactivated Polio Vaccine
Source: Viruses. 2025 Jun 27;17(7):915. doi: 10.3390/v17070915 (PMC12300962; doi:10.3390/v17070915)
Supplement: Supplementary file 1 [file viruses-17-00915-s001.zip › viruses-3689436-supplementary.pdf]

Supplementary Figures

Sample	Values	Mean	Std.Dev.
**Vehicle** Acute 1:300	0.05	0.048	0.001
	0.047		
	0.047		
	0.047		
	0.047		
	0.049		
**Vehicle** Chronic 1:300	0.046	0.047	0.001
	0.046		
	0.046		
	0.046		
	0.047		
	0.048		
**UltraIPV^{TM} Low** Acute 1:300	0.061	0.056	0.003
	0.055		
	0.053		
	0.053		
	0.055		
	0.06		
**UltraIPV^{TM} Low** Chronic 1:300	0.047	0.049	0.004
	0.045		
	0.046		
	0.048		
	0.051		
	0.057		
**UltraIPV^{TM} Med** Acute 1:300	0.053	0.05	0.002
	0.05		
	0.049		
	0.05		
	0.048		
	0.05		
**UltraIPV^{TM} Med** Chronic 1:300	0.049	0.05	0.003
	0.048		
	0.048		
	0.049		
	0.05		
	0.055		
**UltraIPV^{TM} High** Acute 1:300	0.053	0.05	0.002
	0.05		
	0.049		
	0.051		
	0.049		
	0.049		
**UltraIPV^{TM} High** Chronic 1:300	0.047	0.049	0.003
	0.049		
	0.048		
	0.049		
	0.048		
	0.055		
**IPOL^{R}** Acute 1:300	0.052	0.05	0.001
	0.049		
	0.05		
	0.05		
	0.049		
	0.048		
**IPOL^{R}** Chronic 1:300	0.049	0.051	0.004
	0.047		
	0.048		
	0.052		
	0.054		
	0.056		
**Control** No Primary Sera Secondary Only **Peptide Coated**	0.043	0.043	0.003
	0.042		
	0.043		
	0.043		
	0.042		
	0.042		
	0.042		
	0.043		
	0.042		
	0.042		
	0.052		
	0.043		
**Control** **UltraIPV^{TM} High** Acute 1:300 **No Peptide**	0.056	0.053	0.002
	0.053		
	0.052		
	0.054		
	0.052		
	0.051		
**Control** **UltraIPV^{TM} High** Chronic 1:300 **No Peptide**	0.054	0.057	0.003
	0.054		
	0.056		
	0.061		
	0.06		
	0.056		
**Control** **IPOL^{R}** Acute 1:300 **No Peptide**	0.054	0.055	0.004
	0.052		
	0.059		
	0.06		
	0.052		
	0.052		
**Control** **IPOL^{R}** Chronic 1:300 **No Peptide**	0.063	0.058	0.005
	0.054		
	0.054		
	0.057		
	0.064		
	0.054		

Sample	Values	Mean	Std.Dev.
**Vehicle** Acute 1:1000	0.042	0.046	0.003
	0.044		
	0.044		
	0.046		
	0.051		
	0.048		
**Vehicle** Chronic 1:1000	0.044	0.045	0.002
	0.047		
	0.044		
	0.043		
	0.042		
	0.047		
**UltraIPV^{TM} Low** Acute 1:1000	0.045	0.045	0.001
	0.045		
	0.046		
	0.044		
	0.043		
	0.045		
**UltraIPV^{TM} Low** Chronic 1:1000	0.042	0.043	0.001
	0.043		
	0.042		
	0.042		
	0.042		
	0.044		
**UltraIPV^{TM} Med** Acute 1:1000	0.045	0.045	0.002
	0.045		
	0.045		
	0.049		
	0.043		
	0.042		
**UltraIPV^{TM} Med** Chronic 1:1000	0.042	0.043	0.001
	0.042		
	0.043		
	0.043		
	0.044		
	0.045		
**UltraIPV^{TM} High** Acute 1:1000	0.045	0.045	0.001
	0.045		
	0.046		
	0.047		
	0.046		
	0.043		
**UltraIPV^{TM} High** Chronic 1:1000	0.043	0.045	0.001
	0.045		
	0.044		
	0.045		
	0.045		
	0.046		
**IPOL^{R}** Acute 1:1000	0.046	0.046	0.001
	0.046		
	0.046		
	0.046		
	0.048		
	0.045		
**IPOL^{R}** Chronic 1:1000	0.045	0.045	0.001
	0.046		
	0.043		
	0.046		
	0.044		
	0.046		
**Control** No Primary Sera Secondary Only **Peptide Coated**	0.074	0.044	0.009
	0.043		
	0.042		
	0.043		
	0.043		
	0.04		
	0.041		
	0.041		
	0.042		
	0.042		
	0.041		
	0.041		
**Control** **UltraIPV^{TM} High** Acute 1:1000 **No Peptide**	0.064	0.051	0.008
	0.055		
	0.046		
	0.046		
	0.046		
	0.046		
**Control** **UltraIPV^{TM} High** Chronic 1:1000 **No Peptide**	0.045	0.048	0.004
	0.044		
	0.047		
	0.05		
	0.056		
	0.047		
**Control** **IPOL^{R}** Acute 1:1000 **No Peptide**	0.047	0.047	0.002
	0.044		
	0.047		
	0.049		
	0.047		
	0.046		
**Control** **IPOL^{R}** Chronic 1:1000 **No Peptide**	0.044	0.046	0.002
	0.044		
	0.044		
	0.046		
	0.047		
	0.049		

Figure S1. DPI ELISA

A450nm absorbances shown for ELISA assay that was performed to determine whether sera from immunized rats reacted to the DPI peptide required in the inactivation step of *ultra*IPV^{TM} preparation.

PV1 - S					
**Day 38**	**IPOL^{R}**	**UltraIPV^{TM} Low**	**UltraIPV^{TM} Med**	**UltraIPV^{TM} High**	**Vehicle**
**IPOL^{R}**	1	0.9389340	0.9389340	0.9389340	0.0000007
**UltraIPV^{TM} Low**	0.9389339	1	0.9389340	0.9389340	0.0000024
**UltraIPV^{TM} Med**	0.9389339	0.9389340	1	0.9389340	0.0000025
**UltraIPV^{TM} High**	0.9389339	0.9389340	0.9389340	1	0.0000042
**Vehicle**	0.0000007	0.0000020	0.0000030	0.0000040	1
**Day 43**	**IPOL^{R}**	**UltraIPV^{TM} Low**	**UltraIPV^{TM} Med**	**UltraIPV^{TM} High**	**Vehicle**
**IPOL^{R}**	1	0.7610880	0.7610880	0.7610880	0.0008660
**UltraIPV^{TM} Low**	0.7610880	1	0.7610880	0.5977520	0.0095650
**UltraIPV^{TM} Med**	0.7610880	0.7610880	1	0.7610880	0.0023140
**UltraIPV^{TM} High**	0.7610880	0.5977520	0.7610880	1	0.0003570
**Vehicle**	0.0008660	0.0095650	0.0023140	0.0003570	1
**Day 59**	**IPOL^{R}**	**UltraIPV^{TM} Low**	**UltraIPV^{TM} Med**	**UltraIPV^{TM} High**	**Vehicle**
**IPOL^{R}**	1	0.8394640	0.7174200	0.7993170	0.0007280
**UltraIPV^{TM} Low**	0.8394640	1	0.6846440	0.7174200	0.0003610
**UltraIPV^{TM} Med**	0.7174200	0.6846440	1	0.8394640	0.0068310
**UltraIPV^{TM} High**	0.7993170	0.7174200	0.8394640	1	0.0040060
**Vehicle**	0.0007280	0.0003610	0.0068310	0.0040060	1

nOPV2					
**Day 38**	**IPOL^{R}**	**UltraIPV^{TM} Low**	**UltraIPV^{TM} Med**	**UltraIPV^{TM} High**	**Vehicle**
**IPOL^{R}**	1	0.6032810	0.0879507	0.4185860	0.0001800
**UltraIPV^{TM} Low**	0.6032810	1	0.2662205	0.7218410	0.0000088
**UltraIPV^{TM} Med**	0.0879510	0.2662210	1	0.4185860	0.0000000
**UltraIPV^{TM} High**	0.4185860	0.7218410	0.4185863	1	0.0000015
**Vehicle**	0.0001800	0.0000090	0.0000000	0.0000020	1
**Day 43**	**IPOL^{R}**	**UltraIPV^{TM} Low**	**UltraIPV^{TM} Med**	**UltraIPV^{TM} High**	**Vehicle**
**IPOL^{R}**	1	0.7821230	0.5808800	0.5808800	0.0110480
**UltraIPV^{TM} Low**	0.7821230	1	0.7215660	0.7215660	0.0032130
**UltraIPV^{TM} Med**	0.5808800	0.7215660	1	0.9394120	0.0003640
**UltraIPV^{TM} High**	0.5808800	0.7215660	0.9394120	1	0.0003640
**Vehicle**	0.0110480	0.0032130	0.0003640	0.0003640	1
**Day 59**	**IPOL^{R}**	**UltraIPV^{TM} Low**	**UltraIPV^{TM} Med**	**UltraIPV^{TM} High**	**Vehicle**
**IPOL^{R}**	1	0.7785690	0.8458220	0.8458220	0.0055950
**UltraIPV^{TM} Low**	0.7785690	1	0.8458220	0.8458220	0.0004390
**UltraIPV^{TM} Med**	0.8458220	0.8458220	1	0.8792450	0.0020880
**UltraIPV^{TM} High**	0.8458220	0.8458220	0.8792450	1	0.0015820
**Vehicle**	0.0055950	0.0004390	0.0020880	0.0015820	1

PV3 - S					
**Day 38**	**IPOL^{R}**	**UltraIPV^{TM} Low**	**UltraIPV^{TM} Med**	**UltraIPV^{TM} High**	**Vehicle**
**IPOL^{R}**	1	0.0391658	0.3695800	0.3321100	0.0004946
**UltraIPV^{TM} Low**	0.0391660	1	0.2868800	0.3171590	0.0000000
**UltraIPV^{TM} Med**	0.3695800	0.2868799	1	0.8853420	0.0000035
**UltraIPV^{TM} High**	0.3321100	0.3171588	0.8853420	1	0.0000022
**Vehicle**	0.0004950	0.0000000	0.0000040	0.0000020	1
**Day 43**	**IPOL^{R}**	**UltraIPV^{TM} Low**	**UltraIPV^{TM} Med**	**UltraIPV^{TM} High**	**Vehicle**
**IPOL^{R}**	1	0.6666410	0.6666410	0.2360890	0.0063580
**UltraIPV^{TM} Low**	0.6666410	1	0.4861850	0.4269310	0.0014780
**UltraIPV^{TM} Med**	0.6666410	0.4861850	1	0.1147550	0.0240330
**UltraIPV^{TM} High**	0.2360890	0.4269310	0.1147550	1	0.0000120
**Vehicle**	0.0063580	0.0014780	0.0240330	0.0000120	1
**Day 59**	**IPOL^{R}**	**UltraIPV^{TM} Low**	**UltraIPV^{TM} Med**	**UltraIPV^{TM} High**	**Vehicle**
**IPOL^{R}**	1	0.7823140	0.7003850	0.7003850	0.0032360
**UltraIPV^{TM} Low**	0.7823140	1	0.5599150	0.5599150	0.0111080
**UltraIPV^{TM} Med**	0.7003850	0.5599150	1	0.9797990	0.0003240
**UltraIPV^{TM} High**	0.7003850	0.5599150	0.9797990	1	0.0003240
**Vehicle**	0.0032360	0.0111080	0.0003240	0.0003240	1

Figure S2. Binding Antibody ELISA Statistical Analysis

Differences in ELISA absorbances were tested for significance using Kruskal-Wallis and Dunn's test with Benjamini-Hochberg correction for multiple comparison. All corrected p values are shown.

	Organs	Test Animals											
Vehicle	Kidney	0	0	0		0	0	0		0	0	0	0
	Liver	0	0	0		0	0	0		0	0	0	0
	Spleen	0	0	0		0	0	0		0	0	0	0
	Brain	0	0	0		0	0	0		0	0	0	0
	Lungs	0	0	0		0	0	0		0	0	0	0
	Heart	0	0	0		0	0	0		0	0	0	0
	Repro	0	0	0		0	0	0		0	0	0	0
	Injection sites	0	2*	2*		0	0	0		0	0	0	0
UltraIPV™ Low	Kidney	0	0	0	0	0	0	0	0	0	0	0	5*
	Liver	0	0	0	0	0	0	0	0	0	2**	0	0
	Spleen	0	0	0	0	0	0	0	0	0	0	0	0
	Brain	0	0	0	0	0	0	0	0	0	0	0	0
	Lungs	0	0	0	0	0	0	0	0	0	0	0	0
	Heart	0	0	0	0	0	0	0	0	0	0	0	0
	Repro	0	0	0	0	0	0	0	0	0	0	0	0
	Injection sites	0	0	0	0	0	0	0	2***	0	0	0	0
UltraIPV™ Med	Kidney	0	0	0	0	0	0	0	0	0	0	0	0
	Liver	0	0	0	0	0	0	0	0	0	0	0	0
	Spleen	0	0	0	0	0	0	0	0	0	0	0	0
	Brain	0	0	0	0	0	0	0	0	0	0	0	0
	Lungs	1*	0	0	0	0	0	0	0	0	0	0	0
	Heart	0	0	0	0	0	0	0	0	0	0	0	0
	Repro	0	0	0	0	0	0	0	0	0	0	0	0
	Injection sites	0	0	0	0	0	0	2**	0	0	0	0	0
UltraIPV™ High	Kidney	0	0	0	0	0	0	0	0	0	0	0	0
	Liver	0	0	0	0	0	0	0	0	0	0	0	0
	Spleen	0	0	0	0	0	0	0	0	0	0	0	0
	Brain		0	0	0	0	0	0	0	0	0	0	0
	Lungs		0	0	0	0	0	3*	2**	2**	0	1***	1***
	Heart		0	0	0	0	0	0	0	0	0	0	0
	Repro	0	0	0	0	0	0	0	0	0	0	0	0
	Injection sites	1.5****	0	0	0	0	0	0	0	0	0	0	0
IPOL®	Kidney	0	0	0	0	0	0	0	0	0	0	0	0
	Liver	0	0	0	0	0	0	0	0	0	0	0	0
	Spleen	0	0	0	0	0	0	0	0	0	0	0	0
	Brain	0	0	0	0	0	0	0	0	0	0	0	0
	Lungs	0	0	0	0	0	0	0	0	0	0	4*	0
	Heart	0	0	0	0	0	0	0	0	0	0	0	0
	Repro	0	0	0	0	0	0	0	0	0	0	0	0
	Injection sites	0	0	0	0	0	0	0	0	0	0	0	0

Vehicle:
* Mild mononuclear infiltrates

UltraIPV™ Low:
* Nephroblastoma
** Mild periportal infiltrates of mononuclear cells
*** Mild inflammatory infiltrates

UltraIPV™ Med:
* Rare infiltrates of mature neutrophils surrounding vessels
** Mild mononuclear infiltrates

UltraIPV™ High:
* Moderate numbers of mature neutrophils surrounding vessels
** Mild infiltration of mature neutrophils surrounding vessels
*** Rare infiltrates of mature neutrophils surrounding vessels
**** Mild to moderate mononuclear to neutrophilic inflammation

IPOL®:
* Focus of moderate numbers of mature neutrophils surrounding a larger caliber vessel

Abnormality Scoring	
Severity	Points
None	0
Rare	1
Mild	2
Moderate	3
Severe	4
Life-threatening	5

Figure S3. Tissue Histology Scoring

Histological analysis scores represent the severity of tissue sample abnormalities for individual animals in each vaccination group. Annotations and scoring key are shown boxed on the right.
